# 3D Analysis of Upper Limbs Motion during Rehabilitation Exercises Using the Kinect^TM^ Sensor: Development, Laboratory Validation and Clinical Application

**DOI:** 10.3390/s18072216

**Published:** 2018-07-10

**Authors:** Bruno Bonnechère, Victor Sholukha, Lubos Omelina, Serge Van Sint Jan, Bart Jansen

**Affiliations:** 1 Laboratory of Anatomy, Biomechanics and Organogenesis (LABO), Université Libre de Bruxelles, 1050 Brussels, Belgium; vcholouk@ulb.ac.be (V.S.); sintjans@ulb.ac.be (S.V.S.J.); 2 Department of Electronics and Informatics—ETRO, Vrije Universiteit Brussel, 1050 Brussels, Belgium; lomelina@etrovub.be (L.O.); bjansen@etrovub.be (B.J.); 3 International Medical Equipment Collaborative (IMEC), Kapeldreef 75, B-3001 Leuven, Belgium; 4 Department of Applied Mathematics, Peter the Great St. Petersburg Polytechnic University (SPbPU), 195251 Sankt-Peterburg, Russia; 5 Institute of Computer Science and Mathematics, Slovak University of Technology, 81237 Bratislava, Slovakia

**Keywords:** Kinect, validation, assessment, functional evaluation, shoulder, markerless system

## Abstract

Optoelectronic devices are the gold standard for 3D evaluation in clinics, but due to the complexity of this kind of hardware and the lack of access for patients, affordable, transportable, and easy-to-use systems must be developed to be largely used in daily clinics. The Kinect^TM^ sensor has various advantages compared to optoelectronic devices, such as its price and transportability. However, it also has some limitations: (in)accuracy of the skeleton detection and tracking as well as the limited amount of available points, which makes 3D evaluation impossible. To overcome these limitations, a novel method has been developed to perform 3D evaluation of the upper limbs. This system is coupled to rehabilitation exercises, allowing functional evaluation while performing physical rehabilitation. To validate this new approach, a two-step method was used. The first step was a laboratory validation where the results obtained with the Kinect^TM^ were compared with the results obtained with an optoelectronic device; 40 healthy young adults participated in this first part. The second step was to determine the clinical relevance of this kind of measurement. Results of the healthy subjects were compared with a group of 22 elderly adults and a group of 10 chronic stroke patients to determine if different patterns could be observed. The new methodology and the different steps of the validations are presented in this paper.

## 1. Introduction

Since the release of the first version of the Kinect^TM^ sensor for the Xbox 360 (Kinect) at the end of 2010, researchers and clinicians have directly felt the possible potential of this device. Many studies have been done in order to validate this device as a markerless system (MLS) for various uses (e.g., motion analysis, posture analysis, feedback during rehabilitation exercises, etc.) [1,2,3,4,5,6,7].

Interesting results have been found in terms of accuracy (compared to gold standard marker-based systems (MBSs)) and especially in terms of precision (reproducibility has been found higher for Kinect compared to MBS using a Plug-in Gait (PiG) model like the Vicon^TM^ protocol) [3].

3D motion analysis using MBS is considered to be the gold standard for clinical motion analysis, even if several issues have been previously raised and discussed in the literature. The accessibility of MBS is an issue due to the costs of such systems, and therefore only specialized centers can afford them. Furthermore, marker placement, which is time-consuming and is a potential source of error [8], and skin displacement during motion are two recognized problems within the MBS field [9].

Several studies have previously studied the use of the Kinect as an MLS for upper limb evaluation to assess reachable workspace on healthy subjects [10], on patients suffering from fascioscapulohumeral muscular dystrophy [11], and patients with Duchenne muscular dystrophy [12]. Another study compared simple planar motions (shoulder abduction and elbow flexion) and found good correlations with MBS results [4]. Since movement speed control is important in various neurological conditions, others have investigated the ability of the Kinect to detect arm movement speed in healthy subjects, and obtained good results after applying some filtering algorithms [13].

A new generation of Kinect, Kinect for Xbox One (Kinect One), was released in 2014. Research has been done to compare the two generations of Kinect for object detection. The results were better for the second generation of Kinect, especially when the distance between the object and the camera was increased [14]. The use of Kinect One to assess upper limb mobility or function has also been extensively studied. Authors found that 3D evaluation of shoulder ranges of motion were significantly more precise and with narrow limits of agreement than the measurements of trained observers (clinicians), based on the analysis of 1670 measurements [15]. Another study compared the results of the Kinect One, an MBS, and goniometry for range of motion (ROM) and motion smoothness. Kinect One had very good agreement of ROM measurement (r > 0.9) with the 3D motion analysis compared with goniometry. Kinect One also showed a good correlation and agreement of measurement of motion quality parameters compared with the 3D motion analysis [16].

The measurements performed in these studies were not taken at the same time, making comparisons of upper limb evaluation difficult. Other studies compared 3D full body kinematics analysis, mainly during gait–balance and adaptive postural control. Authors found that the accuracy of Kinect One landmark movements was moderate to excellent and depended on movement dimension, landmark location, and the task performed [17]. Another study showed that gait analysis using multiple Kinect One sensors can provide an accurate analysis of human gait compared to MBS [18]. During those two studies, authors successfully recorded motion simultaneously, showing that the infrared signals from the MBS do not provide too much noise and do not influence the results of the Kinect.

Despite these promising results, some issues still need to be solved in order to fully use this MLS in daily clinical practice and for unsupervised remote data collection by patients at home.

Due to the information provided by the Kinect SDK (i.e., a simple skeleton model composed by 20 points for the old Kinect and 25 points for the Kinect One), it is not possible to directly obtain three-dimensional joint orientation poses. Another issue related to motion analysis is the different conventions used (e.g., Euler’s sequences, orientation vector position), making comparison and interpretation of the results difficult [19].

Some solutions have been proposed to increase the quality of the results, such as fusing the data from the Kinect and accelerometers [20], modifying the placement of the sensor according to the type of measurements [21,22], fusing the data from multiple Kinect sensors [18,23], or developing new algorithms for skeleton detection based on raw data [24].

The aim of this study was to present an advanced (PiG, as in the Vicon^TM^ system ) MLS model [25] and a new method for motion analysis based on joint trajectories complementary to joint angles during rehabilitation exercises using a single Kinect camera. Previous studies have shown that the Kinect sensor could be used to follow patients’ evolution during rehabilitation exercises [26,27]. This kind of evaluation, done during the rehabilitation, has many advantages: (i) it is done in the natural environment of the patient (it is known that patients are not exhibiting the same performance when they are wearing underwear in a gait laboratory); (ii) when patients are immersed in the games they are less focused on the motion and on pain and can reach larger amplitudes than when they are asked to perform one particular motion; (iii) it provides time savings; and (iv) it is financially beneficial (the devices are affordable, and since the evaluation is done within the therapy session there is no dual pricing) [28].

The different steps of the development of this method, the laboratory validation (i.e., comparison with a gold-standard optoelectronic device) and clinical validation are presented in this paper. Results of this new method are easier to interpret and could therefore be used in clinics and at home to assess patient status and monitor follow-up.

## 2. Methods

Each frame of the MLS motion data was collected from the original hardware and was available as 3D coordinates of crude approximation of the main human joints (Microsoft Kinect SDK). By piecewise linear connection of those joints, one can develop a stick-based model (i.e., adjacent points are linked together by a line representing human segments) for visualization and motion analysis. The major drawback of this approach is the inability to allow anatomically correct descriptions of the joint angular motion according to current clinical conventions [19]. An algorithm developed to extend the crude model provided by the Kinect containing several steps was previously developed and validated [25].

Each link size can be corrected based on the assumption that the raw stick-based model supplies proper line orientation. Starting from the native thorax stick model, one can substitute the spatial location of extremity joints, and therefore segment size, by processing each link sequentially from the root (e.g., thoracic segment) to the end joint (shoulder, then elbow, and finally wrist joint).

**Figure 1:**
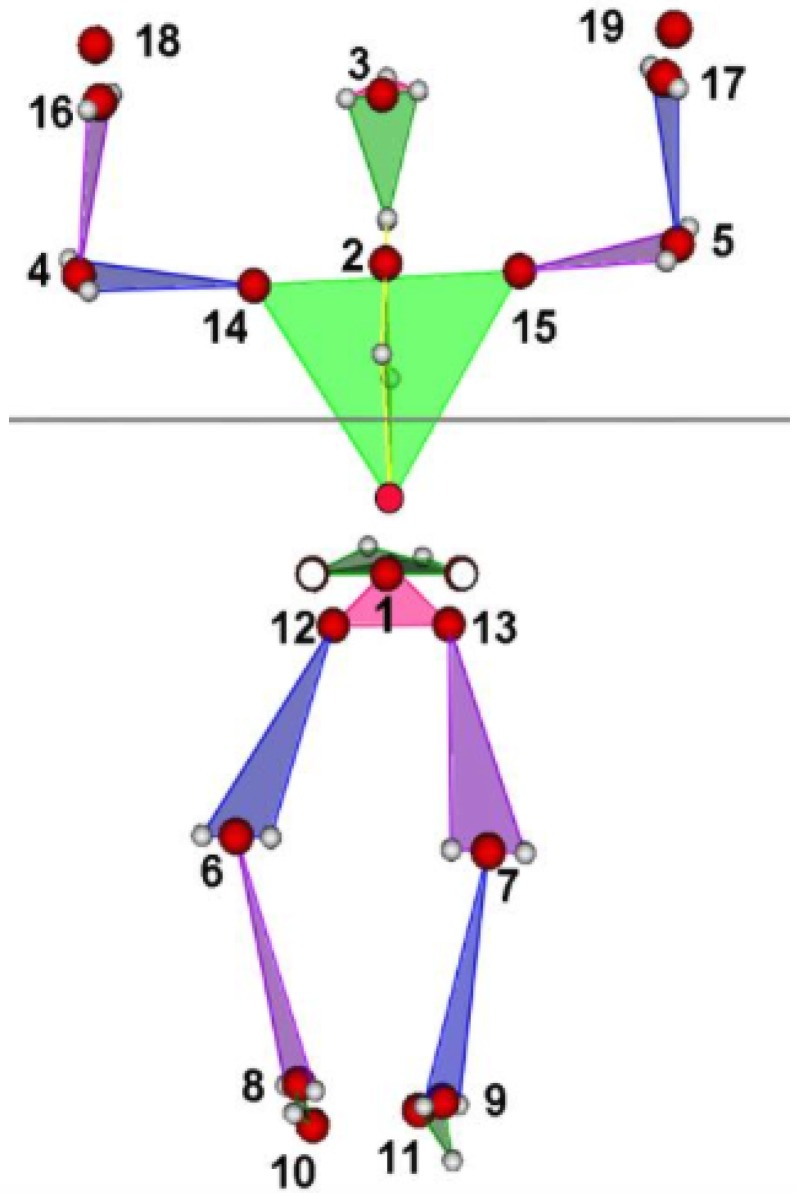
Joint center estimation from the Kinect (red circle), reconstructed Plug-in Gait (PiG)-like data (transparent 34 circles), and 19 local coordinate system origins (indicated by numbers).

In total, 19 local coordinate systems (LCSs), following International Society of Biomechanics (ISB) recommendations [29] for axis orientation, were located in the origins indicated by numbers 1–19 in Figure 1. Then, 33 LCSs origin motions relative to parent LCSs (Table 1) were created.

Each of the 33 trajectory plots were processed to assess different properties of the shape created by hodograph. Similar to Duarte et al., nine parameters (1–9 in Table 2) were estimated directly from the point trajectories [30]. This method was previously developed for balance analysis. Those parameters have been validated to assess dynamic balance during SG rehabilitation exercises [31]. We then extended this 2D analysis system into 3D analysis to get more information about the reaching area of the participants.

All trajectory points were also processed as a point cloud to assess principal and supplementary axes origin and orientation in the parent LCS. The main principal axis corresponded to the maximum eigenvalue. Then, the second axis was defined as perpendicular to the plane of the first axis and the radius vector of the principal axes origin. The last axis direction was the right hand perpendicular to the first two. The size of each axis was defined by min/max points distance estimated from projection on axis the trajectory cloud.

Then, 32 (numbered from 10 to 41) additional trajectory shape definition parameters were evaluated (Table 2). Those 41 parameters were computed using the following equations.

Trajectories data were defined in LCS by a sequence of *N* points with frequency *f* (e.g., f=30 s${}^{-1}$) by

(1) $$p_{i}=\left[p_{ix},p_{iy},p_{iz}\right],i=1,\ldots ,N.$$

The point instantaneous absolute velocity value (||…|| is the Euclidean norm) and the total velocity (1×N) matrix are given by

(2) $$v_{i}=\left|\right|\left[v_{ix},v_{iy},v_{iz}\right]\left|\right|,$$

(3) $$V=[v_{1},\ldots ,v_{N}].$$

The current length of the trajectory (travel) corresponds to $L_{N}$:

(4) $$L_{i}=\sum_{k=1}^{i-1}\left|\right|p_{k+1}-p_{k}\left|\right|,i=2,\ldots ,N.$$

The scalar motion parameters for the hodograph velocity are obtained with

(5) $$V_{Mean}=\frac{1}{N}\sum_{k=1}^{N}v_{k},$$

(6) $$V_{std}={\left(\frac{1}{N-1}\sum_{k=1}^{N}{\left(v_{k}-V_{MN}\right)}^{2}\right)}^{\frac{1}{2}},$$

(7) $$V_{Max}=max\left(V\right).$$

The total area of the trajectory reached by each joint is given by Equation (8), where $S_{i},i=1,\ldots ,N-1$ is the area of the triangle defined by three points $\left[o,p_{i},p_{i+1}\right]$:

(8) $$\Delta_{N}=\sum_{i=1}^{N-1}S_{i}.$$

The angle between two rays $\left[p_{i},p_{i+1}\right]$ is obtained by Equation (9), where $h_{i}=2S_{i}/\left|\right|p_{i}\left|\right|$:

(9) $$\alpha_{i}=arcsin\left(\frac{h_{i}}{\left|\right|p_{i+1}\left|\right|}\right).$$

From there, the total angular travel (in degrees) is obtained with

(10) $$\Phi_{N}=\frac{180}{\pi }\sum_{i=1}^{N-1}\alpha_{i}.$$

The angular velocity instant value and the total angular velocity (1×N−1) matrix are given by Equations (11) and (12), respectively

(11) $$\omega_{i}=\frac{180}{\pi }f\alpha_{i}$$

(12) $$\Omega =[\omega_{1},\ldots ,\omega_{N-1}]$$

From Equation (12), the mean, standard deviation, and maximum angular velocity are easily obtained $\left(\Omega_{Mean},\Omega_{std},\Omega_{Max}\right)$.

The mean (central) point of the cloud is given by

(13) $$p_{Mean}=\frac{1}{N}\sum_{k=1}^{N}p_{k},$$

and the centered point coordinate is

(14) $$q_{i}=p_{i}-p_{Mean}.$$

The instantaneous inertia matrix is obtained with Equation (15), and the total cloud inertia matrix by Equation (16):

(15) $$I_{i}=Eq_{i}q_{i}^{T}-q_{i}^{T}q_{i},E=diag\left(1\right),$$

(16) $$I=\sum_{i=1}^{N}I_{i}.$$

Then, using singular value decomposition, a main first principal axis is obtained

(17) $$G_{1}={\left[G_{1x},G_{1y},G_{1z}\right]}^{T},||G_{1}\left|\right|=1.$$

Two additional orthogonal axes can be obtained using Equations (18) and (19), where *c* is a normalized (unit) vector with $C_{x},C_{y},C_{z}$ projections:

(18) $$c=\frac{c}{\left||c|\right|},\tilde{c}=\left|\begin{array}{ccc} 0 & -c_{z} & c_{y}\\ c_{z} & 0 & -c_{x}\\ -c_{y} & c_{x} & 0 \end{array}\right|,$$

(19) $$G_{2}={\tilde{p}}_{Mean}G_{1}/\left|\right|{\tilde{p}}_{Mean}G_{1}\left|\right|,$$

(20) $$G_{3}={\tilde{G}}_{1}G_{2}.$$

$\tilde{c},\tilde{p}$, and $\tilde{G}$ are skew-symmetric matrix representation for using in matrix shape of vector cross product.

A (3×3) orientation matrix is obtained with Equation (20), and a (N×3) projection matrix of $q_{1}$ on its axis with Equation (21):

(21) $$G=[G_{1},G_{2},G_{3}],$$

(22) $$Q_{G}={\left[q_{1},\ldots ,q_{N}\right]}^{T}G.$$

The minimal and maximal points on orthogonal axis (size (1×3)):

(23) $$Q_{Min}=\underset{i=1,\ldots ,N}{min(Q_{G_{ij}})},j=1,2,3\left(X,Y,Z\right),$$

(24) $$Q_{Max}=\underset{i=1,\ldots ,N}{max(Q_{G_{ij}})},j=1,2,3\left(X,Y,Z\right).$$

For each axis number i=1,2,3, the two end point positions (parameters 19–30 for i=1,2):

(25) $$B_{1i}=p_{Mean}+Q_{Min_{i}}G_{i}^{T},$$

(26) $$B_{2i}=p_{Mean}+Q_{Max_{i}i}G_{i}^{T}.$$

The angle of view (parameters 12 and 13 for i=1,2):

(27) $$\Phi_{Bi}=\left(180/\pi \right)arccos(B_{1i}B_{2i}/\left(|\right|B_{1i}\left|||\right|B_{2i}||)).$$

The size of the axis (parameters 14 and 15 for i=1,2):

(28) $$L_{Bi}=\left|\right|B_{1i}-B_{2i}\left|\right|.$$

The surface area of the rhomboid defined by the end points of the first and second axes (parameter 10) is given by Equation (29):

(29) $$S_{mid}=L_{B1}L_{B2}/2.$$

The volume of the diamond defined by six end points is finally obtained (parameter 11):

(30) $$Vol=S_{mid}L_{B3}/3.$$

Parameters 31–37 (Table 2) are obtained using point fitting by sphere. Parameters 38–41 are obtained using Delaunay triangulation and convex hull functions in Matlab.

**Figure 2:**
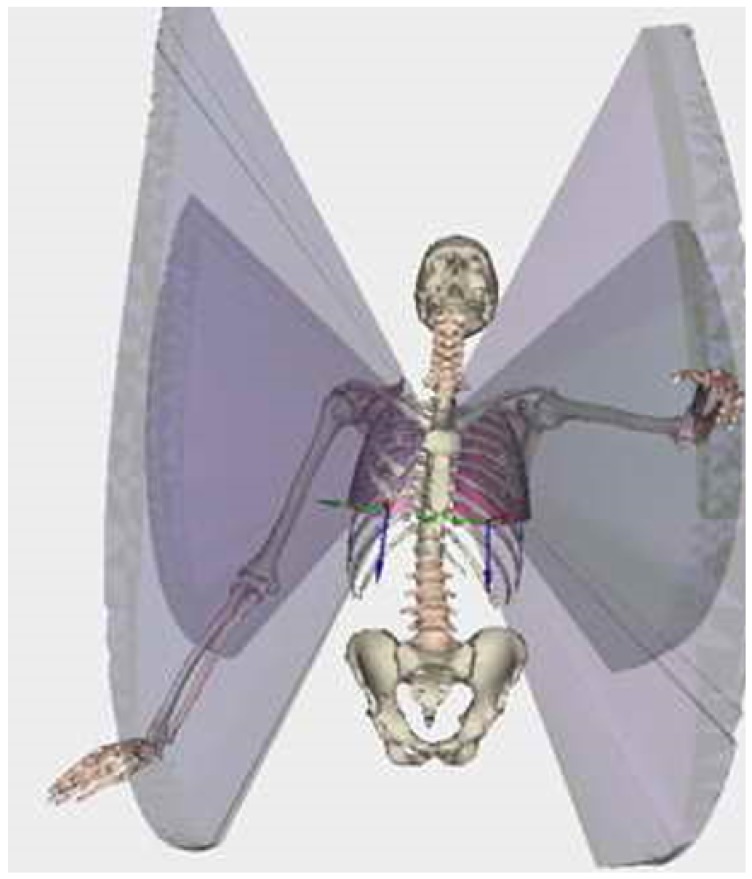
Example of the visualization of results obtained from the rehabilitation game. Visualization is performed here using LHPFusionBox for a limited set of parameters (i.e., volumetric parameters for wrist and elbow by point trajectory triangulation). The reachable volume is clearly visible, but no direct quantification (i.e., score) is available.

Example of the different scores related to the reaching area are presented in Figure 2.

Using this method, up to 697 variables can be processed (41 parameters × 17 joints). An intuitive and easy-to-interpret visualization tool must be developed to present the results and allow comparison between patients and control or to perform longitudinal patient follow-up.

This score can be based on single or multiple joints analysis.

For single joints, the total amount of analyzed joints is $N_{\phi }$. For each single joint *i*, the angular values are $\phi_{i}^{k}=\left[\phi_{ix}^{k},\phi_{iy}^{k},\phi_{iz}^{k}\right],i=1,\ldots ,N_{\phi },k=c,pt$, where the *k* index corresponds to control (*c*) or patient (pt) data. The joint angle dimensionless score values can be defined as $s_{ij}^{\phi }=\phi_{ij}^{c}/\phi_{ij}^{pt},i=1,\ldots ,N_{\phi },j=x,y,z$, and the joint mean weighted score as $S_{\phi i}=\left(w_{ix}^{\phi }s_{ix}^{\phi }+w_{iy}^{\phi }s_{iy}^{\phi }+w_{iz}^{\phi }s_{iz}^{\phi }\right)/\left(w_{ix}^{\phi }+w_{iy}^{\phi }+w_{iz}^{\phi }\right),i=1,\ldots ,N_{\phi }$, where $w_{ij}^{\phi }$ are weight factors. Total body joint mean weighted ($W_{i}^{\phi }$) score, from selected joints $i\in i_{\phi }$, can finally be derived as $C_{\phi }=\sum_{i\in i_{\phi }}W_{i}^{\phi }S_{\phi i}/\sum_{i\in i_{\phi }}W_{i}^{\phi }$ .

This approach can be extended in the case of multiple joints data analysis based on trajectories analysis. In the presented model for upper limb assessment, the total number of trajectories ($N_{j}$) is 17, and the total number of parameters for each trajectory ($N_{p}^{j}$) is 41. Parameter values can be obtained with $p_{ij}^{k},i=1,\ldots ,N_{p}^{j},j=1,\ldots ,N_{j},k=c,pt$ for a selected parent joint *j*, parameter number *i*, and control (*c*) or patient (pt) parameter values. Then, a dimensionless parameter score can be defined as $s_{ij}^{p}=p_{ij}^{c}/p_{ij}^{p},i=1,\ldots ,N_{p}^{j},j=1,\ldots ,N_{J}$, and a trajectory *j* mean weighted ($W_{ij}^{p}$) score, from a selected joint $i\in I_{p}$, can be defined as $S_{pj}=\sum_{i\in I_{p}}W_{ij}^{p}S_{ij}^{p}/\sum_{i\in I_{p}}W_{ij}^{p},j=1,\ldots ,N_{J}$. The total body trajectory-based analysis mean weighted score ($W_{j}^{p}$), from selected trajectories $j\in J_{p}$, can be finally derived as $C_{p}=\sum_{j\in J_{p}}W_{j}^{p}S_{pj}/\sum_{j\in J_{p}}W_{j}^{p}$.

**Figure 3:**
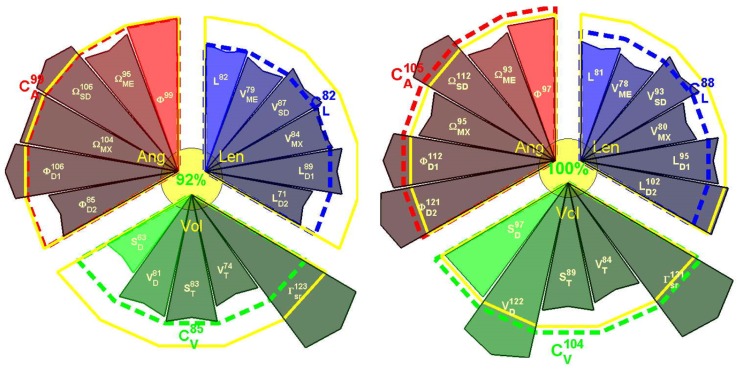
Example of scoring visualization for Right and Left upper limbs from selected motion scoring for one trial of a stroke patient. The scoring was obtained from the 17 parameters defined above. Parameters are grouped by angular (Ang, in red), length (Len, in blue), and volumetric (Vol, in green) properties. Yellow contour corresponds to 100% (healthy group comparison). Parameter sign values are explained in Table 2. Scores for each group and total scores are depicted near the sector of the group and in the center, respectively.

An example of this visualization tool is presented in Figure 3.

## 3. Laboratory Setting Validation

### 3.1. Participants

Forty healthy adults (24 ± 6 years old, 172 ± 8 cm height, 68 ± 10 kg weight, 23 ± 3 kg/m${}^{2}$ BMI, 18 women) were recruited to participate in this study. This study was approved by the Ethical Committee of the Erasme Hospital (EudraCT/CCB : B406201215142), and written informed consent was obtained from all subjects prior to their participation.

### 3.2. Material

The MLS sensor (Kinect) was placed on a tripod 1.5 m above the floor. Subjects stood 2.5 m from the camera; this distance was found to provide optimal results in a previous study [3]. Subjects were in underwear to allow reliable placement of the markers for the MBS analysis taking place simultaneously. Experiments took place in a motion analysis laboratory.

**Figure 4:**
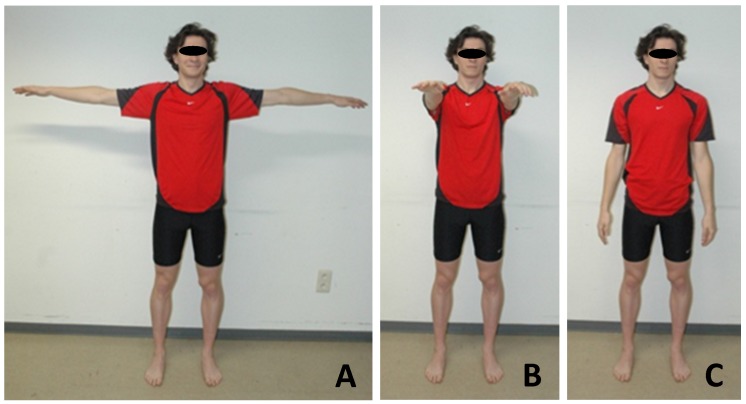
The three calibration poses: (**A**) “T-pose”; (**B**) “Wide pose”; (**C**) “Upright pose”.

Prior to motion analysis, the subjects were asked to stand still in anatomical position facing the MLS camera. Subjects were then asked to maintain three different poses (3 s for each of the poses; see Figure 4) before recording the motion in order to calibrate the MLS data processing pipeline.

MBS data were simultaneously collected from a state-of-the-art stereophotogrammetric system (Vicon, 8 MXT40s cameras, Vicon Nexus software, frequency: 90 Hz) that tracks the spatial trajectories of the reflective markers set on the subjects. A modified Plug-in Gait (PiG) model was adopted. In addition tothe usual PiG markers, markers were set on the medial epicondyle of the humeral and femoral bones. Thirty-four markers were positioned by the same observer during the entire study. The image frame rate used was equal to 30 fps for the MLS. MLS data were collected with a laptop (Sony Vaio SVF15323CXB, 1.6 GHz Intel Core i5-4200U, 6 GB DDR3L SDRAM, 750 GB (5400 rpm) SATA Hard Drive).

### 3.3. The Serious Games

**Figure 5:**
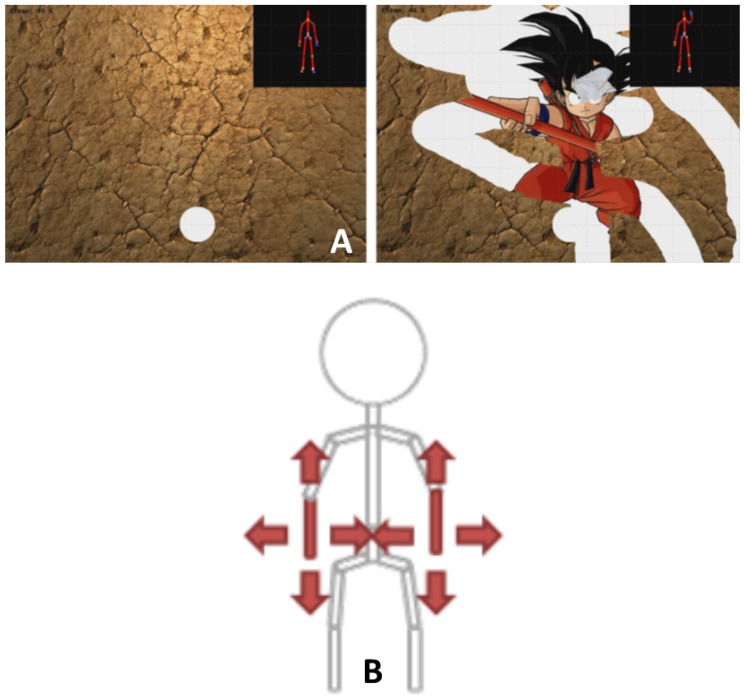
(**A**) Screenshot of the especially developed serious games used in the study; (**B**) Illustration of the motion required to control the game.

Participants played one mini-game that was especially developed for physical rehabilitation: the Wipe Out [32] (Figure 5). The player has to clean the screen covered with mud using a tissue controlled by mediolateral and inferior–superior displacements of the wrist relative to the trunk. Participants were asked to play three repetitions of the games. Motions were simultaneously recorded with the MBS and the MLS.

### 3.4. Data Processing and Statistics

The different scores and parameters described above were computed for the two devices. The mean of the results of three repetitions of the games were computed for statistical analysis. Normality of the data was checked graphically (histogram, boxplot, and qplot) and Shapiro–Wilk test. Mean values and standard deviations were calculated. Discrepancies between the MBS and the different versions of MLS were tested using Pearson’s correlation coefficient (R). The reproducibility coefficient (RCP=1.96 × STD) and the coefficient of variation (CV=STD/Mean) were expressed as percentages. Statistics and data processing were done with MATLAB and Statistics Toolbox Release 2016a (The Mathworks, Inc., Natick, Massachusetts, United States).

### 3.5. Results of the Laboratory Validation

Due to space restriction only some results are presented and will be discussed.

For upper limb analysis, up to 328 parameters can be obtained (4 joints × 2 sides × 41 parameters)

Results of the seven selected parameters for the relative displacements of elbow relative to shoulder (“shoulder”, points 4 and 5 in Figure 1) and wrist relative to shoulder (“wrist”, points 16 and 17 in Figure 1) for right and left sides are presented in Table 3.

All the parameters, except the angular velocity (mean R = 0.51 for the four joints), presented good correlation between results of the MLS and the MBS. On the other hand, the best results in terms of correlation, RCP, and CV were obtained for the velocity (expressed in m/s).

For both shoulders and wrists, better results were obtained for the total length of the trajectory, the total angle, and the mean velocity.

Although good correlations were found for parameters related to the reaching area (i.e., volume, sphere, and surface), lower RCP and CV were found for them.

The next step of the study was to determine if those parameters are sensitive enough to discriminate healthy subjects from patients.

## 4. Validation in Clinical Environment

### 4.1. Participants

Three groups of subjects and patients were tested in order to evaluate the clinical relevance of the newly developed evaluation method and scores:

- *Adults*: Sixteen healthy young adults (results of the laboratory validation were used)
- *Elderly*: Seventeen patients (79±5 years old) hospitalized in a geriatric department were included in the study. This study was approved by the local ethical committee of Erasme Hospital (Eudract: B406201628246), and informed consent was obtained from the patients prior to their participation.
- *Stroke*: 10 patients with chronic stroke (73±8 years old) participated in this study. This study was approved by the ethical committee of Erasme Hospital (EudraCT: B406201526116), and informed consent was obtained from the patients prior to their participation.

### 4.2. Material

The same methodology as for the laboratory setting validation was used. Patients were in underwear so that clothing did not interfere with skeletal detection. Experiments were done in the hospital rooms, in contrast to the other protocol, the patients were not equipped with reflective markers. This situation is more natural than the evaluation performed in the gait lab and close to the daily clinics and rehabilitation.

### 4.3. Data Processing and Statistics

Each participant played three repetitions of the games. The different scores and parameters described above were computed for the three groups. The mean of the results of three repetitions of the games were computed for statistical analysis. Normality of the data was checked using the Shapiro—Wilk test. Mean values and standard deviations were calculated. One-way analysis of variance (ANOVA) was used to compared the groups, and post-hoc analysis was done using the Bonferroni procedure.

### 4.4. Results of the Validation in the Clinical Environment

Mean results of the three groups and statistics are presented in Table 4. The same parameters as during the clinical validation are presented.

Concerning the shoulders, no statistically significant difference was found for the length, but highly significant differences were found for both the total angle, the velocity for young adults and elderly individuals, and stroke patients. The only parameters that could differentiate the three groups, based on relative motion of the elbow relative to the shoulder, was the volume of the sphere.

Concerning the wrists, statistically significant differences were found for the length and the velocity between young adults, elderly, and stroke patients. For the volume, significant differences were found only between young adults and elderly individuals. The surface and the total angles presented statistically significant differences between the three groups.

## 5. Discussion

3D evaluation of the upper limbs is still a complex task in clinics, due to non-cyclic motions, various degrees of freedom, different conventions for presenting the results or processing methods [19,29], etc.

The availability of the Kinect sensors coupled to the development and use of serious games in physical rehabilitation [33] offers a new perspective for long-term evaluation and follow-up during rehabilitation. Microsoft stopped manufacturing the Kinect in 2015 and the Kinect One in 2017. Therefore, other 3D cameras (e.g., Orbbec Astra Pro^TM^, Asus Xtion sensors^TM^) or other affordable devices (e.g., multiple RGBD cameras [34]) could be used instead of the Kinect.

It is indeed possible to track and analyze motions performed by patients during serious games exercises [35]. However, there are still some problems to solve in order to get relevant information to provide feedback for both patients and clinicians. Compared to the most-used motion analysis in clinics (the gait analysis), the data collected during serious games rehabilitation exercises are usually longer (mini-games are approximately one minute, gait analysis is only focusing on a few steps), non-cyclic (gait cycles are normalized by step), and involve free motions (patients need to perform a task but they can use different strategies (e.g., shoulder or elbow)). Therefore, it is not possible to average and normalize the motions performed by the patients, and analyzing only the ranges of motion is too restrictive to summarize one-to-two minute exercises.

Two solutions are possible to obtain relevant information from the rehabilitation exercises:

The first is to analyze the performance of the patients within the games [36]: time required to finish, number of successes, failures, precision, etc. Although those parameters are relevant in clinics, they are only an indirect indicator of the status of the patients. Direct indicators (i.e., biomechanic and functional analysis) should be obtained by analyzing the motions performed by the patients and extracting clinically relevant information.

The second solution, presented in this paper, is to analyze the trajectories performed by the patients and extract relevant information about speed, total displacement, and reaching area.

First, the results obtained with the MLS were compared with gold-standard MBS. Good agreements were found between the MLS and MBS for the different studied parameters, especially for the speed-related parameters (m/s) and the reaching area (Table 3). Gross and fine motor controls are complex tasks involving many different components of the central and peripheral nervous systems [37]. Important natural alterations occur during a lifetime: a slow maturation of all the components during childhood to acquire gross and later fine motor control [38]; then, physiological declines of motor functions are observed starting around 60 years old [39]. Speed of motion is among the most clinically relevant information in aging [40] and in various neurological diseases (e.g., stroke [41]). However, not only the speed of motion is important in clinics [42]—without gross and fine motor control, patients cannot independently perform activities of daily living [43]. Therefore, this analysis must be coupled with an accuracy assessment [36].

Reaching area and other related parameters informing about the autonomy of patients are popular in rehabilitation and occupational therapy, since this is a good indicator of the independence of the patients [44]. The variables about the volume reached or the area swept in the average plane of motion were highly correlated between both devices (Table 3).

During the second part of this study, we tested the system with elderly and stroke patients to determine if the scores could differentiate the three groups. Concerning aging, we observed a decrease of velocity and of the reaching area, which is coherent with the physiology of aging [39]. Concerning the comparison between elderly and stroke patients, the results must be interpreted carefully because of the small sample size. This part of the study was a proof of concept to evaluate the possibility of such kind of techniques. Larger studies are needed to determine if different motor patterns can be found depending on various pathologies.

One of the possible issues related to this method is that up to 328 parameters can be obtained for upper limbs analysis. It is thus not possible for clinicians or patients to analyze all those parameters. Two problems must be solved before the system can be used in clinics: data reduction/selection and data visualization.

Two methods can be used to determine the more relevant parameters.

The first is based on expert’s (i.e., clinician’s) opinion and expertise. According to the pathology, they select what they think is the most appropriate and relevant [45].

The second approach is to use statistical methods. Principal component analysis can be used to select the most discriminant parameters for each population (if the sample size is large enough). Clustering or other machine learning methods can be used to determine the most relevant parameters to detect differences between healthy subjects and patients [46].

Both methods have advantages and disadvantages. The advantage of expert-based selection (supervised) is that the clinicians (the final users of this solution) are choosing parameters that they understand and are meaningful. The weak point of this method is that they are probably missing plenty of relevant information because of the number of new parameters that they are unaware of. Concerning the unsupervised method, it is the opposite situation: all of the data is analyzed without prior clinical assumptions and therefore parameters will be selected that are relevant from a statistical point of view but which may be difficult to interpret and/or understand for the clinicians. This gap between the clinic and the development of new methods and technology is becoming increasingly important, and special attention must be paid to it in order to continue developing useful technologies [47]. A mixed approach between clinically oriented selection of the data (experts’ opinion) and machine learning methods must be encouraged in order to have solutions that can be used in daily clinics.

Due to financial constraints and the lack of access to clinicians, the time in front of the patient during consultation is continuously decreasing [48]. In this particular context, rapid and easy-to-interpret visualization tools must be developed. An alternative visualization of the scoring (compared to Table 3 and Table 4) is presented in Figure 3.

Selected parameters (*n =* 17) for visualization are grouped per angular (Ang, *n =* 6), volumetric (Vol, *n =* 5), and length (Len, *n =* 6) characteristics. The reference value of the score is 100%, and is indicated by the yellow circle. Score values of the parameters in the range [±100%] are presented inside each sector, and the radius of the sector is proportional to the score value. Mean scores are presented for each group, and the total score from the 17 parameters is depicted in the yellow circle in the center. The main representative results for parameters from angle, length, and volume are plotted in a star diagram (Figure 3).

In this example, results for both limbs are compared with reference values of healthy subjects and expressed in percentage.

In the case of asymmetric pathologies (e.g., hemiplegia), results of the affected limb can be compared with the healthy one [49].

Future work will focus on the selection of the best parameters and including other relevant parameters such as the smoothness of the motion using normalized jerk in order to assess the quality of the exercises [50].

## 6. Conclusions

Quantified 3D functional evaluation of the upper limb is still a challenge for clinicians. This evaluation is particularly important in order to best guide treatment and revalidation in order to guarantee optimal results and thus improve patient autonomy.

From a technological point of view, the major advantage of this new method is the frame-by-frame straightforward calculation of its 34 additional points from the crude skeleton captured by MLS in order to evaluate and visualize full 3D data in real time. Point trajectory analysis is usually used for converting marker tracks to six degrees of freedom (DoFs) link motion if at least three link related markers are available [51]. This is a well-defined way of representing motion kinematics, but it requires some specific knowledge about orientation and translation representation in global or/and local coordinate systems. This knowledge (ISB conventions, biomechanical background) has been incorporated in the model to enrich MLS data.

In addition to the optimization algorithm, several parameters were processed based on the trajectories performed by the patients. Further studies are needed to select which parameters are the most relevant to perform functional evaluation and long-term follow-up during the rehabilitation. Results of the analysis are presented for intuitive and easy-to-understand interpretation for both patients and clinicians thanks to the user-friendly visualization interface.

From the rehabilitation point of view, the innovative approach presented in this paper of combining revalidation exercises with functional evaluation offers many advantages: saving time and money, patients are immersed in the game and can therefore perform more repetitions but also more natural movements because they do not have the impression of being evaluated, evaluation is automatic and objective, it does not require the presence of a clinician, measurements can be made in the patient’s ecological environment on a frequent basis, patients can directly visualize the evolution of their results from session-to-session, etc.

The proposed new scoring system to perform functional assessment coupled to rehabilitation exercises has been validated. Therefore, results of this kind of evaluation could be used to monitor patients and to perform long-term follow-up during rehabilitation thanks to the visualization interface. These tools can be useful for both patients and clinicians.

## Figures and Tables

**Table 1:** Relative coordinate systems topology for upper limbs assessment, origin segments (child local coordinate systems) are expressed relative to parent local coordinate systems. 0 corresponds to a global coordinate system. Point numbers are presented in Figure 1.

Motion	1	4	5	14	15	16	17	18	19	20	21	22	23	24	25	26	27
Child	1	4	5	14	15	4	5	16	17	18	19	16	17	18	19	18	19
Parent	0	2	2	2	2	14	15	14	15	14	15	4	5	4	5	16	17

**Table 2:** List of parameters evaluated for trajectory analysis.

Parameter	Unit	Value	Equation
1	m	Total length of the trajectory	4
2	deg	Total angle of the trajectory (hodograph)	10
3–5	m/s	Mean, std, and max of the hodograph velocity	5–7
6–8	deg/*s*	Mean, std, and max of hodograph angular velocity	12
9	deg/*s*	Mean hodograph angular velocity from parameter 3 and mean radius	From 5
10	cm${}^{2}$	Square of cross-sectional rhomboid, defined by first and second axes	29
11	cm${}^{3}$	Volume of two pyramids (diamond) constructed from three axes end points	30
12, 13	deg	Angles of view of two main axes from parent local coordinate system (LCS) origin	27
14, 15	mm	Size of the two main axes	28
16–18	mm	Position of principal axes origin in the parent LCS
19–30	mm	Position of the end points of the first two axes in parent LCS	25, 26
31	deg	Radius of the cloud fitting by sphere	Point fitting by sphere
32	mm	Distance between LCS origin and sphere centre	Point fitting by sphere
33	mm	Mean residual of sphere fitting	Point fitting by sphere
34	mm	std residual of sphere fitting	Point fitting by sphere
35–37		Position in LCS of the fitted sphere centre	Point fitting by sphere
38, 39	mm${}^{2}$, mm${}^{3}$	Triangulated surface area and conic volume (with vertex in the LCS origin)	Delaunay triangulation and convex hull functions
40, 41	sr	Solid angle (steradians (sr)) of the sphere and triangulated surface	Delaunay triangulation and convex hull functions

**Table 3:** Comparison between the optoelectronic (marker-based system, MBS) and the Kinect (markerless system, MLS) systems. R is the Pearson coefficient of correlation, RCP is the reproducibility coefficient expressed in percent, and CV is the coefficient of variation.

Joint	Variables	R	RCP (%)	CV
Right Shoulder	Length (mm)	0.71 *	45	32
	Angle (deg)	0.56 *	32	41
	Velocity (m/s)	0.96 *	31	18
	Angular velocity (deg/s)	0.50	71	47
	Volume (mm${}^{3}$)	0.73 *	65	45
	Sphere (cm${}^{3}$)	0.98 *	63	40
	Surface (mm${}^{2}$)	0.83 *	52	53
Left Shoulder	Length (mm)	0.72 *	35	38
	Angle (deg)	0.58 *	46	41
	Velocity (m/s)	0.94 *	31	19
	Angular velocity (deg/s)	0.56	67	44
	Volume (mm${}^{3}$)	0.64 *	54	55
	Sphere (cm${}^{3}$)	0.96 *	55	38
	Surface (mm${}^{2}$)	0.98 *	60	51
Right Wrist	Length (mm)	0.71 *	35	38
	Angle (deg)	0.88 *	21	26
	Velocity (m/s)	0.95 *	33	16
	Angular velocity (deg/s)	0.51	58	75
	Volume (mm${}^{3}$)	0.79 *	57	40
	Sphere (cm${}^{3}$)	0.97 *	66	56
	Surface (mm${}^{2}$)	0.98 *	53	48
Left Wrist	Length (mm)	0.68 *	39	34
	Angle (deg)	0.92 *	16	24
	Velocity (m/s)	0.89 *	28	15
	Angular velocity (deg/s)	0.47	57	46
	Volume (mm${}^{3}$)	0.72 *	41	49
	Sphere (cm${}^{3}$)	0.88 *	55	45
	Surface (mm${}^{2}$)	0.95 *	47	43

* Statistically significant correlation (*p* < 0.05).

**Table 4:** Mean (std) results of the studied variables for the three groups, *p*-Values are the results of the  ANOVA.

Joint	Variables	Adults	Elderly	Stroke	*p*-Value
Right shoulder	Length (mm)	3.81 × 10${}^{7}$ (3.7 × 10${}^{7}$)	3.64 × 10${}^{7}$ (9.1 × 10${}^{6}$)	5.71 × 10${}^{7}$ (1.89 × 10${}^{7}$)	0.21
	Angle (deg)	2.95 × 10${}^{4}$ (1.3 × 10${}^{3}$)	1.12 × 10${}^{4}$ (7.2 × 10${}^{3}$) ${}^{\alpha }$	1.11 (5.2 × 10${}^{3}$) ${}^{\beta }$	<0.001
	Velocity (m/s)	0.21 (0.09)	0.12 (0.06) ${}^{\alpha }$	0.10 (0.4) ${}^{\beta }$	<0.001
	Angular velocity (deg/s)	315 (283)	403 (775)	329 (221)	0.71
	Volume (mm${}^{3}$)	6.21 × 10${}^{11}$ (2.1 × 10${}^{11}$)	8.12 × 10${}^{11}$(1.6 × 10${}^{11}$)	6.94 × 10${}^{11}$ (1.53 × 10${}^{11}$)	0.12
	Sphere (cm${}^{3}$)	3.52 × 10${}^{11}$ (2.1 × 10${}^{11}$)	7.68 × 10${}^{11}$ (2.4 × 10${}^{11}$) ${}^{\alpha }$	4.85 × 10${}^{11}$ (1.4 × 10${}^{11}$) ${}^{\beta ,\gamma }$	0.04
	Surface (mm${}^{2}$)	6.25 × 10${}^{11}$ (2.5 × 10${}^{11}$)	2.31 × 10${}^{12}$ (8.9 × 10${}^{11}$)	3.62 × 10${}^{11}$ (3.1 × 10${}^{11}$)	0.07
Left Shoulder	Length (mm)	3.88 × 10${}^{7}$ (1.6 × 10${}^{6}$)	2.96 × 10${}^{7}$ (3.4 × 10${}^{7}$)	4.38 × 10${}^{7}$ (1.0 × 10${}^{8}$)	0.64
	Angle (deg)	2.72 × 10${}^{4}$ (1.2 × 10${}^{3}$)	1.23 × 10${}^{4}$ (7.2 × 10${}^{3}$) ${}^{\alpha }$	1.2 × 10${}^{4}$ (5.1 × 10${}^{3}$) ${}^{\beta }$	<0.001
	Velocity (m/s)	0.19 (0.06)	0.13 (0.06) ${}^{\alpha }$	0.10 (0.04) ${}^{\beta ,\gamma }$	<0.001
	Angular velocity (deg/s)	271 (251)	344 (230)	345 (317)	0.61
	Volume (mm${}^{3}$)	6.13 × 10${}^{11}$ (4.6 × 10${}^{11}$)	1.17 × 10${}^{12}$ (1.1 × 10${}^{12}$)	7.71 × 10${}^{11}$ (1.3 × 10${}^{11}$)	0.13
	Sphere (cm${}^{3}$)	3.81 × 10${}^{11}$ (8.4 × 10${}^{10}$)	1.18 × 10${}^{12}$ (2.3 × 10${}^{12}$) ${}^{\alpha }$	4.18 × 10${}^{11}$ (1.30 × 10${}^{11}$) ${}^{\beta ,\gamma }$	0.03
	Surface (mm${}^{2}$)	6.27 × 10${}^{11}$ (1.4 × 10${}^{11}$)	9.8 × 10${}^{11}$ (1.9 × 10${}^{11}$)	3.36 × 10${}^{11}$ (5.8 × 10${}^{10}$)	0.06
Right Wrist	Length (mm)	3.77 × 10${}^{7}$ (3.1 × 10${}^{7}$)	5.58 × 10${}^{7}$ (5.2 × 10${}^{7}$) ${}^{\alpha }$	5.9 × 10${}^{7}$ (7.5 × 10${}^{7}$) ${}^{\beta }$	0.04
	Angle (deg)	3.13 × 10${}^{7}$ (3.2 × 10${}^{7}$)	3.89 × 10${}^{7}$ (8.43 × 10${}^{6}$)	7.91 × 10${}^{7}$ (1.2 × 10${}^{7}$) ${}^{\beta }$	0.03
	Velocity (m/s)	0.23 (0.09)	0.13 (0.07) ${}^{\alpha }$	0.10 (0.04) ${}^{\beta }$	<0.001
	Angular velocity (deg/s)	280 (226)	351 (242)	323 (311)	0.58
	Volume (mm${}^{3}$)	7.01 × 10${}^{11}$ (1.2 × 10${}^{11}$)	1.12 × 10${}^{12}$ (1.5 × 10${}^{11}$) ${}^{\alpha }$	5.67 × 10${}^{11}$ (8.44 × 10${}^{11}$)	0.04
	Sphere (cm${}^{3}$)	5.81 × 10${}^{11}$ (7.1 × 10${}^{10}$)	8.41 × 10${}^{11}$ (9.0 × 10${}^{10}$)	6.11 × 10${}^{11}$ (5.5 × 10${}^{10}$)	0.21
	Surface (mm${}^{2}$)	5.92 × 10${}^{11}$ (1.2 × 10${}^{10}$)	1.33 × 10${}^{12}$ (2.8 × 10${}^{11}$) ${}^{\alpha }$	2.71 × 10${}^{11}$ (6.4 × 10${}^{11}$) ${}^{\beta ,\gamma }$	0.03
Left Wrist	Length (mm)	3.69 × 10${}^{7}$ (3 × 10${}^{7}$)	5.57 × 10${}^{7}$ (4.2 × 10${}^{7}$) ${}^{\alpha }$	5.29 × 10${}^{7}$ (4.3 × 10${}^{7}$) ${}^{\beta }$	0.04
	Angle (deg)	3.11 × 10${}^{7}$ (3.3 × 10${}^{7}$)	3.64 × 10${}^{7}$ (6.1 × 10${}^{7}$) ${}^{\alpha }$	6.33 × 10${}^{7}$ (7.6 × 10${}^{7}$) ${}^{\beta ,\gamma }$	0.03
	Velocity (m/s)	0.31 (0.14)	0.12 (0.07) ${}^{\alpha }$	0.11 (0.05) ${}^{\beta }$	<0.001
	Angular velocity (deg/s)	281 (246)	384 (314)	294 (245)	0.22
	Volume (mm${}^{3}$)	6.84 × 10${}^{11}$ (1.1 × 10${}^{11}$)	1.52 × 10${}^{12}$ (2.65 × 10${}^{11}$) ${}^{\alpha }$	5.91 × 10${}^{11}$ (9.1 × 10${}^{11}$)	0.03
	Sphere (cm${}^{3}$)	5.89 × 10${}^{11}$ (6.4 × 10${}^{11}$)	5.44 × 10${}^{11}$ (6.1 × 10${}^{11}$)	4.12 × 10${}^{11}$ (5.4 × 10${}^{11}$)	0.42
	Surface (mm${}^{2}$)	4.61 × 10${}^{11}$ (9.8 × 10${}^{10}$)	1.41 × 10${}^{12}$ (2.6 × 10${}^{11}$) ${}^{\alpha }$	3.42 × 10${}^{11}$ (2.6 × 10${}^{11}$) ${}^{\beta ,\gamma }$	0.02

${}^{\alpha }$ Statistically significant difference between Adults and Elderly after Bonferroni correction. ${}^{\beta }$ Statistically significant difference between Adults and Stroke after Bonferroni correction. ${}^{\gamma }$ Statistically significant difference between Elderly and Stroke after Bonferroni correction.

## Abbreviations

The following abbreviations are used in this manuscript:

MLS:	Markerless system
MBS:	Marker-based system
LCS:	Local coordinate system
RCP:	Reproducibility coefficient
CV:	Coefficient of variation
